# Allelopathic and autotoxic effects of sorghum extract and residues on seed behavior, and morphological, physiological, and biochemical responses of several plants

**DOI:** 10.1038/s41598-026-39434-2

**Published:** 2026-02-16

**Authors:** Faezeh Shahmohammadi, Mehrdad Abdi, Ali Faramarzi, Jalil Ajalli, Hassan Nourafcan

**Affiliations:** 1 Department of Agronomy and Plant Breeding, Mi.C., Islamic Azad University, Miyaneh, Iran; 2 Department of Horticultural Science, Mi.C., Islamic Azad University, Miyaneh, Iran

**Keywords:** Allelopathy, Autotoxicity, Oxidative stress, PEG-6000, Sorghum residues, Total phenolic compounds, Biochemistry, Physiology, Plant sciences

## Abstract

This study investigated the allelopathic potential of sorghum (*Sorghum bicolor* L.) by applying aqueous extracts (0, 2, 4, 6, and 8%), root residues, and burned root residues to eight crop species (sorghum, corn, wheat, barley, sunflower, rapeseed, alfalfa, and cowpea) under PEG-6000–induced drought stress (20% PEG) using a two-phase design (in vitro and greenhouse). In the petri dish experiment, sorghum derivatives caused clear, concentration-dependent reductions in germination indices, seedling growth, biomass accumulation, and biochemical attributes, including photosynthetic pigments, proline, soluble carbohydrates, and antioxidant enzyme activities (CAT, SOD, and APX). Alfalfa and cowpea showed the highest sensitivity to the combined allelopathic and osmotic stress and were excluded from the greenhouse assay. During the greenhouse phase, species-specific tolerance patterns emerged: sorghum showed the greatest resilience to root residue treatments under drought conditions, while the remaining crops displayed varying degrees of susceptibility. Overall, the findings demonstrate strong dose-responsive allelopathic effects of sorghum across laboratory and greenhouse conditions, highlighting its potential for sustainable weed management and crop rotation systems, while underscoring the importance of interspecific variation in plant tolerance to drought stress.

## Introduction

Plants often face stressful conditions (such as drought, salt, heavy metals, and others), which severely impair seed germination, seedling establishment, and overall crop productivity^1,2^. At the same time, chemical interactions among plants, known as allelopathy, represent a distinct type of stress^3,4^. Allelopathy refers to the ability of plants to release chemical signals and bioactive secondary metabolites (allelochemicals) through root exudates, leaf leachates, or decomposing residues, which can negatively affect the growth, development, and germination of neighboring plants, and in some cases, the producing plant itself, termed autotoxicity^5–7^. This phenomenon has received increasing attention for its potential applications in sustainable agriculture and environmentally friendly weed management^8–10^.In this regard, the release of allelochemicals into the surrounding environment via root exudation or the decomposition of plant residues has been shown to inhibit the growth and establishment of undesirable plant species, which has been documented for some crops, including maize, sorghum, wheat, barley, and rye^11^. On the other hand, as global reliance on synthetic herbicides raises concerns about ecological degradation, herbicide resistance, soil health deterioration, and food safety, natural plant-based approaches offer promising alternatives^9,12,13^. Among allelopathic species, sorghum (Sorghum bicolor) stands out due to its remarkable production of potent allelochemicals, including sorgoleone, dhurrin, and a wide array of phenolic acids^14,15^. These compounds are actively exuded from sorghum roots, leaves, and residues, and have been shown to inhibit the germination and growth of numerous weed species^16,17^. Empirical evidence indicates that incorporation of sorghum residues into the soil, either as mulch, green manure, or surface litter, can significantly reduce the biomass of problematic weeds such as *Bromus diandrus* (great brome) and *Silybum marianum* (milk thistle), with suppression rates exceeding 80% under controlled conditions^17^. Despite the known suppressive effects, the allelopathic performance of sorghum residues is highly dependent on their physical and chemical status^18–20^. Fresh or intact plant residues slowly release allelochemicals over time through microbial decomposition and moisture leaching, contributing to prolonged inhibitory effects^21,22^. Conversely, burned plant residues, common in some agricultural systems, may alter the chemical profile by degrading thermolabile compounds like sorgoleone, while potentially enriching the soil with thermally stable phenolics and ash-based nutrients. Yet, the comparative allelopathic efficacy of these two residue forms remains insufficiently studied, particularly concerning species-specific responses and autotoxic feedbacks.

One of the most interesting phenomena is also reported as autotoxicity or self-inhibition, where a plant’s allelochemicals inhibit its growth or regeneration. For sorghum, this is an emerging concern, and some experiments have revealed that certain high-sorgoleone cultivars experience suppressed germination and seedling vigor when exposed to their residues, especially under high concentrations or continuous cropping systems^23,24^. In some cases, inhibitory concentrations of sorghum extract have been shown to reduce its early growth metrics, indicating a potential risk to stand establishment and productivity.

Despite the growing body of knowledge, critical questions remain, particularly regarding how intact versus burned sorghum residues differ in their allelopathic and autotoxic effects and whether their respective leachates exhibit distinct biochemical profiles. Addressing these gaps is essential for optimizing sorghum-based weed suppression strategies while preventing unintended autotoxicity in sorghum cropping systems. Therefore, this study first evaluated, under laboratory conditions, the allelopathic and autotoxic effects of sorghum aqueous extracts (0, 2, 4, 6, and 8%), intact and burned root residues, and PEG-induced osmotic stress on germination dynamics, early growth, and biochemical responses of multiple crop species, including sorghum, corn, wheat, barley, alfalfa, sunflower, rapeseed, and cowpea. We hypothesized that sorghum derivatives, alone or in combination with PEG-mediated drought stress, exert differential and interactive effects on seed behavior and key physiological and biochemical traits, with the magnitude of response being species-dependent. Based on the laboratory outcomes, the most tolerant plant species were subsequently transferred to the greenhouse to simulate field-like conditions. In the greenhouse phase, intact and burned sorghum root residues (5% v/v) were incorporated into the potting soil at four incorporation times (at sowing time, and one, two, and three months before sowing) to assess further their effects on the investigated traits, under conditions designed to mimic field-like conditions. Clarifying these interactions is expected to provide mechanistic insights into sorghum residue management and improve the sustainability of sorghum-based farming systems.

Overall, given the documented allelopathic potential of sorghum-derived metabolites, their variable effects on germination-related indices, and the additional constraints imposed by PEG-induced osmotic stress, it was hypothesized that sorghum extracts and root residues, together with PEG-induced drought stress in Petri dish conditions, exert interactive and differential autotoxic and allelopathic influences capable of modulating early seed behavior and key morphological, physiological, and biochemical traits. Accordingly, this study aimed to (i) elucidate the extent to which sorghum aqueous extracts, soil-incorporated residues, and PEG-mediated osmotic stress (individually and in combination) affect germination dynamics and seedling vigor of selected crop species; (ii) characterize their impacts on chlorophyll status, oxidative responses, and metabolic adjustments under both normal and osmotic-stress conditions; and (iii) compare autotoxic versus interspecific allelopathic patterns to determine whether the magnitude and nature of these responses are species-dependent and stress-dependent. Testing this hypothesis is expected to provide mechanistic insights into sorghum’s allelopathic interactions and clarify how water-deficit conditions modulate allelopathic and autotoxic effects, thereby contributing to improved management of sorghum residues in cropping systems.

## Materials and methods

In this study, the responses of eight plant species were initially evaluated under Petri dish conditions. This allowed assessment of germination indices, biochemical parameters, and early growth traits, and enabled the selection of species tolerant to stress imposed by different levels of sorghum extracts and residues under two levels of polyethylene glycol (PEG).

### Experimental design and replications

The Petri dish and greenhouse experiments were conducted using a factorial arrangement based on a completely randomized design (CRD) with three replicates per treatment. In the Petri dish experiment, eight plant species (including sorghum, corn, wheat, barley, sunflower, rapeseed, alfalfa, and cowpea) were evaluated for germination indices, biochemical parameters, and early growth attributes. Treatments consisted of seven levels of extracts and residues, including control (distilled water), 2%, 4%, 6%, 8% extracts, root residues, and burned root residues, under two levels of PEG, including control (no PEG) and 20% PEG. Each replicate consisted of 25 sterilized seeds placed on a single filter paper in a Petri dish, uniformly moistened with the corresponding treatment solutions for comparison. Finally, the least tolerant species (alfalfa and cowpea) were excluded from subsequent greenhouse experiments.

In the greenhouse experiment, six tolerant plant species (Sorghum, Corn, Wheat, Barley, Sunflower, and Rapeseed) were grown in individual pots containing standardized soil. Treatments included two levels of root residues (Control and Root residues at a 5% volumetric ratio) applied at four different times before planting: T0 (at planting), T1 (one month before planting), T2 (two months before planting), and T3 (three months before planting). Each treatment was replicated three times, and all replicates were managed under identical conditions to ensure consistency and comparability of results. This factorial design enabled the evaluation of the effects of species, extracts/residues, PEG, and root residue application on germination, growth, and biochemical traits across both experimental setups.

Before conducting the experiment, sorghum extracts, residues, and burned residues were also analyzed for physicochemical properties and bioactive compound contents (Table 1). Similarly, the soil used for greenhouse cultivation was analyzed for its physical and chemical properties, and nutrients were adjusted according to laboratory recommendations (Table 2).

**Table 1:** Chemical composition of extracts, root residues, and burned rot residues of sorghum in the present study.

Compound/bioactive substance	Sorghum aqueous extract (mg g^-1^ FW)	Root residues (mg g^-1^ DW)	Burned root residues (mg g^-1^ Ash)
Sorgoleone	0.22	0.91	–
p-Coumaric acid	0.38	0.74	0.06
Ferulic acid	0.61	1.22	0.09
Vanillic acid	0.19	0.45	–
Benzoic acid	0.31	0.67	0.05
Chlorogenic acid	0.15	0.19	–
Total Flavonoids	1.87	2.46	–
Total Phenolics	4.85	8.21	1.37
Tannins	0.73	1.28	0.10
Cellulose	–	31.6	–
Hemicellulose	–	24.3	–
Lignin	–	14.8	–
K (Potassium)	0.32	0.61	7.45
Ca (Calcium)	0.18	0.37	4.12
Mg (Magnesium)	0.09	0.22	2.76
Si (Silicon)	–	0.18	3.68
pH	6.2	6.7	9.3
EC (mS/cm)	1.1	1.8	7.9

**Table 2:** Physicochemical properties and fertilizer recommendations of soil samples for the present greenhouse experiments.

Soil texture	pH	Ec (dS.m^-1^)	OC (%)	N (%)	P (mg.kg^-1^)	K (mg.kg^-1^)	Cu (ppm)	CaCO_3_ (%)
Silty-Clay	7.76	1.54	0.858	0.08	12.2	495	5.1	5
		Nutrient recommendations						
	DAP (Kg ha^-1^)		N (Kg ha^-1^)		P_2_O_5_ (Kg ha^-1^)		K_2_O (Kg ha^-1^)	
	120		150		60		–	

### Measurements

In general, the traits germination percentage (GP), mean time to germination (MTG), germination speed index (GSI), seed vigor indices (SVI1, SVI2), shoot length (SL), root length (RL), root-to-shoot ratio (RSR), aerial dry weight (ADW), root dry weight (RDW), chlorophyll a (Chl a), chlorophyll b (Chl b), total chlorophyll (Chl T), carotenoids (CAR), soluble carbohydrates (SC), leaf proline content (LPC), total phenolic compounds (TPC), and enzymatic activities of catalase (CAT), ascorbate peroxidase (APX), and superoxide dismutase (SOD) were measured in Petri dish conditions. Eventually, the more tolerant species were selected for subsequent greenhouse evaluation. In the greenhouse experiment, the selected species were further evaluated under soil-based growth conditions to assess their physiological and biochemical responses. Specifically, chlorophyll a (Chl a), chlorophyll b (Chl b), total chlorophyll (Chl T), carotenoids (CAR), leaf proline content (LPC), soluble carbohydrates (SC), total phenolic compounds (TPC), and enzymatic activities of catalase (CAT), superoxide dismutase (SOD), and ascorbate peroxidase (APX) were estimated. These traits were analyzed to determine how the selected species respond to a more realistic cultivation environment and to evaluate the extent to which greenhouse conditions can simulate potential field-level performance in future studies.

#### Germination percentage (GP)

Twenty-five sterilized seeds were first placed into 25 × 150 mm Petri dishes lined with a single filter paper soaked with the studied treatments (i.e., 2, 4, 8, and 12 dS.m-1) in three replicates. The germinated seeds were then counted after ten days to estimate the germination percentage (GP) using the following formula, in which n and N refer to the total number of germinated seeds and the total number of seeds, respectively.

1 $$GP (\% ) = \frac{n}{N} \times 100$$

#### Mean time to germination (MTG)

The mean time to germination (MGT) index is defined as a measurement of the rate/time spread of germination and is shown as an average time assumed for seeds to germinate, which is usually calculated by the following equation:

2 $$\text{MTG }\mathrm{=}\frac{\left(\mathrm{N}1\mathrm{T}1\right)+ \left(\mathrm{N}2\mathrm{T}2\right)+\dots +\left(\mathrm{NiTi}\right)}{n}$$

In which N, T, and n refer to the number of seeds germinated on day x, the time from the beginning of the germination test in terms of days, and the total number of germinated seeds.

#### Germination Speed Index (GSI)

The GSI was estimated by dividing the number of daily germinated seeds by the corresponding days. Therefore, the SG trait was calculated according to Eq. 3, in which higher values indicate faster germination.

3 $$\text{GSI } = \frac{n1}{d1}\text{ + }\frac{n2}{d2}+ \frac{n3}{d3}+ \frac{ni}{di}$$

where “n and d” are the number of germinated seeds and the number of days, respectively.

#### Seed Vigor Index (SVI)

The seed vigor index-I and seed vigor index-II were calculated according to the method described by Abdul-Baki & Anderson^25^ as follows:

4 $${\text{Seed vigor index}} - {\text{I }} = {\text{ Standard Germination }}\left( \% \right) \, \times {\text{ Seedling length }}\left( {{\mathrm{cm}}} \right)$$

5 $${\text{Seed vigor index}} - {\text{II }} = {\text{ Standard Germination }}\left( \% \right) \, \times {\text{ Seedling dry weight }}\left( {{\mathrm{mg}}} \right)$$

#### Shoot length (SL) and Root length (RL)

Shoot and root length values were manually measured using a scaled ruler (cm) from the top of the seed tip to the end of the shoot and from the bottom of the seed tip to the end of the root, respectively.

#### Root/Shoot Ratio (RSR)

The root/shoot ratio (RSR) was measured for each treatment by dividing the root dry weight to the shoot.

#### Aerial dry weight (ADW) and Root dry weight (RDW)

To measure ADW and RDW traits, aerial and root parts of each treatment were selected, and their fresh weight was measured after careful washing and drying using a digital scale (0.001 g). After that, the samples were placed in a special paper pocket and placed into an oven (Shimadzu model) at 70 °C for 48 h. Then, both ADW and RDW traits were weighed and recorded separately.

#### Pigment Assay (Chl a, b, Total, and CAR)

Chlorophyll a, chlorophyll b, and total chlorophyll contents were determined spectrophotometrically. Approximately 0.5 g of fresh leaf tissue was homogenized in 80% (v/v) aqueous acetone and centrifuged at 4000 rpm for 10 min. The residue was re-extracted to ensure complete pigment recovery, and the combined extracts were adjusted to a final volume of 10 mL. Chlorophyll contents were calculated according to Porra et al.^26^ using absorbance values at 663.6 and 646.6 nm, and expressed on a fresh weight basis (mg.g^-1^ FW) as follows:

6 $$Chlorophyll a ({mg g}^{-1}FW)=\frac{{V}_{extract}\times [12.25 \times {A}_{663.6}-2.55 \times {A}_{646.6}]}{{1000 \times W}_{tissue}}$$

7 $$Chlorophyll b ({mg g}^{-1}FW)=\frac{{V}_{extract}\times [20.31 \times {A}_{663.6}-4.91 \times {A}_{646.6}]}{{1000 \times W}_{tissue}}$$

8 $$Total chlorophyll({mg g}^{-1}FW)=\frac{{V}_{extract}\times [7.34 \times {A}_{663.6}+17.76 \times {A}_{646.6}]}{{1000 \times W}_{tissue}}$$

Carotenoid contents were also determined following Lichtenthaler^27^, with absorbance measured at 470 nm and concentration calculated using the following equation:

9 $$Carotenoids ({mg g}^{-1}FW)=\frac{{V}_{extract}\times [\left(1000\times A470)-(1.82\times Chl.a)-(85.02\times Chl.b\right)]}{{1000 \times W}_{tissue} \times 198}$$

#### Leaf Proline Content (LPC)

Proline concentration was measured using the Bates et al.^28^ protocol with a slight modification, wherein free proline reacts with ninhydrin at 100°C to form a toluene-soluble red chromophore. Briefly, 0.5 g of fresh leaf tissue was firstly homogenized in 10 mL of 3% sulfosalicylic acid, then filtered with Whatman filter paper No.1, and 2 mL of the filtrate was combined with 2 mL acid-ninhydrin reagent and 2 mL glacial acetic acid. The mixture was incubated for 60 min (at 100 °C), cooled on ice for 30 min, and extracted with 4 mL toluene after vortexing (15–20 s). Following the formation of two phases at room temperature, the upper (colored) toluene layer was separated, and its absorbance was read at 520 nm using a spectrophotometer. Finally, the proline content was estimated based on the standard curve at concentrations of 0, 50, 100, 200, and 250 µM as follows:

10 $$LPC \left( {\mu {\mathrm{M}}.{\mathrm{g}}^{ - 1} FW} \right) = \frac{{\left[ {\frac{{\left( {\mu g.{\mathrm{ml}}^{ - 1} {\mathrm{proline}}\; \times \;{\text{ ml toluene}}} \right)}}{{115.5 \mu {\mathrm{g}}.\mu \;{\mathrm{mole}}^{ - 1} }}} \right]}}{{\left[ {\frac{{\left( {{\mathrm{g}} {\mathrm{sample}}} \right)}}{5}} \right]}}$$

#### Soluble carbohydrates (SC) content

SC was quantified using the anthrone method described by Amerian et al.^29^. Therefore, ethanol-extracted samples (1.0 g of fresh leaf tissue ground in 5 mL ethanol 80%) were first centrifuged at 4°C for 15 min. Then 0.1 mL of the obtained extract was mixed with 3 mL of anthrone reagent (containing 150 mg in 100 mL of 72% H₂SO₄). After that, it was heated for 10 min, and cooled immediately on ice until 23 °C following the formation of the colored substance. Finally, the absorbance was measured at 625 nm using a spectrophotometer (Cary 100 Conc).

#### Catalase activity (CAT)

CAT activity was assayed per Chaoui et al.^30^ in 50 mM potassium phosphate buffer (pH 7.0, 1 mM EDTA, 2% PVPP, 4°C). The reaction mixture (25 mM phosphate buffer, 10 mM H₂O₂, enzyme extract) was monitored at 240 nm, with activity calculated using the extinction coefficient (ε = 0.036 mM⁻^1^cm⁻^1^; Eq. 6), where A, ε, b, and c represent the recorded absorbance, extinction coefficient, cuvette diameter, and H_2_O_2_ concentration, respectively. It should be noted that enzyme extraction involved homogenizing 1 g tissue in 3 mL of 50 mM phosphate buffer (pH 7.2, 1 mM EDTA, 1 mM PMSF, 1% PVP), followed by centrifugation (14,000 × g, 15 min, 4 °C).

11 $$A=\varepsilon bc$$

### Superoxide Dismutase (SOD) Activity

SOD activity was spectrophotometrically assessed via the protocol described by Giannopolitis and Ries^31^ with modifications. Here, the reaction mixture (with 3 mL final volume) contained 40 mM K-phosphate buffer (pH 7.8), 10 mM methionine (2-amino-4-(methyl-thio)-butyric acid), 33 µM nitro-blue tetrazolium (NBT), 3.3 µM riboflavin, 0.66 mM ethylenediamine tetraacetic acid (EDTA), and 0.17% enzyme extract. In the next step, test tubes were irradiated under 15-W fluorescent lights for 30 min, and the absorbance was read at 560 nm for each solution.

#### Ascorbate Peroxidase (APX) Activity

APX activity was determined according to the method of Nakano and Asada^32^ by monitoring ascorbate oxidation at 290 nm (ε = 2.8 mM⁻^1^cm⁻^1^) in a 2 mL reaction system (250 mM phosphate buffer [pH 7.0], 0.1 mM EDTA, 0.5 mM ascorbate, 1.2 mM H₂O₂). The APX activity was expressed in units per mg protein.

#### Statistical Analysis

Data were analyzed using SAS 9.4, and means were compared using the least significant difference (LSD) test at *p* < 0.05. Figures were also prepared with Microsoft Excel.

## Results

### Petri dish experiments

#### Germination-related indices

ANOVA results (Table 3) revealed that seed types (ST), extracts/residues (E/R), and polyethylene glycol (PEG) levels had significant main effects on germination percentage (GP), mean time to germination (MTG), germination speed (SG), and seed vigor indices (SVI1, SVI2) at *p* < 0.01. Interactions between E/R × PEG significantly influenced GP, MTG, SVI1, and SVI2 (*p* < 0.01), while the SG trait was affected by the ST × PEG interaction (*p* < 0.05). Other higher-order interactions were not significant. These results indicate that both allelochemical treatments and drought stress (PEG) strongly affected germination performance, with species-specific responses.

**Table 3:** Analysis of variance (mean squares) of germination and morpho-physiological indices in petri culture conditions.

Sources	df	GP	SG	MTG	SVI1	SVI2	SL	RL	RSR	ADW	RDW
Seed types (ST)	7	1087.07**	0.52**	0.13**	1,372,532.69**	2340.4**	21.62**	8.64**	0.003ns	0.0269**	0.0159**
Extracts/Residues (E.R)	6	2454.41**	2.28**	0.32**	2,280,134.78**	4267.71**	28.88**	14.01**	0.003ns	0.0435**	0.0306**
Polyethylene glycol (PEG)	1	2720.05**	0.47**	0.48**	2,270,013.84**	4033.88**	21.6**	10.01**	0.01ns	0.0212**	0.0247**
ST × E.R	42	17.24ns	0.05ns	0.012ns	10,166.21ns	19.21ns	0.52ns	0.18ns	0.005ns	0.0005ns	0.0006ns
ST × PEG	7	9.41ns	0.14*	0.016ns	11,533.60ns	28.49ns	0.85*	0.05 ns	0.004ns	0.0019*	0.0013*
E.R × PEG	6	896.6**	0.11ns	0.13**	402,371.9**	669.69**	1.57**	0.49**	0.002ns	0.0021*	0.0005ns
ST × E.R × PEG	42	14.92ns	0.10ns	0.007ns	12,642.55ns	30.89ns	0.4ns	0.2ns	0.004ns	0.0006ns	0.0006ns
Error (E)	224	23.91	0.06	0.008	12,157.69	28.37	0.39	0.16	0.004	0.0007	0.0005
CV (%)		6.56	6.88	6.46	7.58	8.38	6.22	4.33	8.31	5.8	6.34

ns: non-significant; ** and * significant at *p* < 0.01 and *p* < 0.05, respectively

According to the mean comparisons of the ST treatment (Table 4), sorghum seeds exhibited the highest values for germination percentage (GP, 82.09%), seed vigor index 1 (SVI1, 1763.27), and seed vigor index 2 (SVI2, 77.05), as well as the lowest mean time to germination (MTG, 1.33 days). In contrast, cowpea seeds showed the lowest GP, SVI1, and SVI2 values (64.38%, 1102.73, and 49.74, respectively) and the highest MTG (1.52 days). Alfalfa seeds had intermediate values for GP, SVI1, and SVI2 (73.62%, 1414.62, and 62.17, respectively). Statistical comparisons indicated that sorghum seeds outperformed other species, with GP increases of 3.93–21.57%, SVI1 increases of 12.66–37.46%, and SVI2 increases of 13.17–35.44% compared to Corn, Wheat, Barley, Sunflower, Rapeseed, Alfalfa, and cowpea. Additionally, Sorghum germinated significantly faster than all other species, with MTG reductions of 3.01–14.29% relative to the other seeds (Table 4).

**Table 4:** Mean comparisons (mean squares) of the germination indices in terms of plant treatment.

Sources	GP (%)	MTG (days)	SVI1	SVI2
Sorghum	82.09 a	1.33 c	1763.27 a	77.05 a
Corn	78.86 b	1.38 b	1539.98 b	66.9 b
Wheat	73.81 b	1.4 b	1432.16 cd	62.35 cd
Barley	74.19 b	1.4 b	1457.63 cd	63.12 cd
Sunflower	74.19 b	1.39 b	1456.79 cd	62.37 cd
Rapeseed	75.33 b	1.37 b	1470.8 c	64.52 c
Alfalfa	73.62 b	1.4 b	1414.62 d	62.17 d
Cowpea	64.38 c	1.52 a	1102.73 e	49.74 e
LSD	2.1	0.04	47.42	2.29

Means sharing the same superscripts are not significantly different from each other at *p* < 0.05.

The interaction effects of extracts/residues (E/R) × polyethylene glycol (PEG) on germination-related indices revealed strong concentration-driven inhibition and the highest values for GP (89.5%), SVI-1 (1895.15), and SVI-2 (81.87) were observed for the control (no E/R) × control (no PEG) treatment. Conversely, the minimum MTG (1.25 days) was also recorded for this interaction (Table 5).

**Table 5:** Effects of E/R × PEG interaction on some germination indices.

Sources		GP (%)	MTG (days)	SVI1	SVI2
Extracts/Residues (E/R)	Polyethylene glycol (PEG)				
Control	Control	89.50 a	1.25 d	1895.15 a	81.87 a
	20%	85.67 b	1.29 d	1745.37 b	77.08 b
2%	Control	74.17 c	1.41 b	1572.13 cd	68.66 cd
	20%	76.17 c	1.38 bc	1566.51 cd	67.94 d
4%	Control	76 c	1.36 c	1612.83 c	70.42 c
	20%	75.83 c	1.39 bc	1522.02 d	66.7 d
6%	Control	75.33 c	1.38 bc	1416.8 e	62.05 e
	20%	57.67 d	1.62 a	1002.79 g	45.44 g
8%	Control	75.50 c	1.40 bc	1425.38 e	61.72 e
	20%	57 d	1.61 a	996.03 g	43.22 g
Burned root residues	Control	75.33 c	1.37 bc	1420.34 e	61.87 e
	20%	75 c	1.37 bc	1421.55 e	61.67 e
Root residues	Control	76 c	1.36 c	1415.96 ef	62.36 e
	20%	74.67 c	1.39 bc	1353.6 f.	58.39 f.
**LSD**		1.51	0.04	44.35	1.65

Means sharing the same superscripts are not significantly different from each other at *p* < 0.05.

**Fig. 1:**
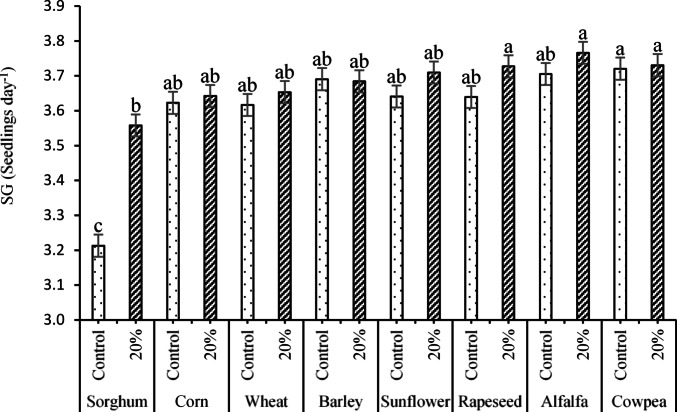
The interaction effects of ST × PEG on the SG trait Means sharing the same superscripts are not significantly different from each other at *p* < 0.05.

Considering the significant effects of the ST × PEG interaction on SG, the data (Fig. 1) indicated that the minimum SG value (3.21 seedlings day^-1^) was recorded for sorghum seeds × no PEG (control), representing the fastest germination. In contrast, the highest SG values (indicating slower germination) were observed for alfalfa × 20% PEG (3.77 seedlings day^-1^), cowpea × 20% PEG (3.73 seedlings day^-1^), and rapeseed × 20% PEG (3.73 seedlings day^-1^), respectively.

#### Morpho-physiological traits

ANOVA results for the morphological and physiological traits (Table 3) indicated that the main treatments and the E/R × PEG interaction significantly affected SL at *p* < 0.01, while the ST × PEG interaction also had a significant effect at *p* < 0.05. The RL index was influenced by the main effects of ST, E/R, and PEG, as well as the E/R × PEG interaction at *p* < 0.01. In addition, ADW showed significant differences for all main treatments at *p* < 0.01 and for both ST × PEG and E/R × PEG interactions at *p* < 0.05. Similarly, RDW was significantly affected by ST, E/R, and PEG (*p* < 0.01) and by the ST × PEG interaction (*p* < 0.05). In contrast, root/shoot ratio (RSR) was not significantly influenced by either main treatments or interactions.

**Fig. 2:**
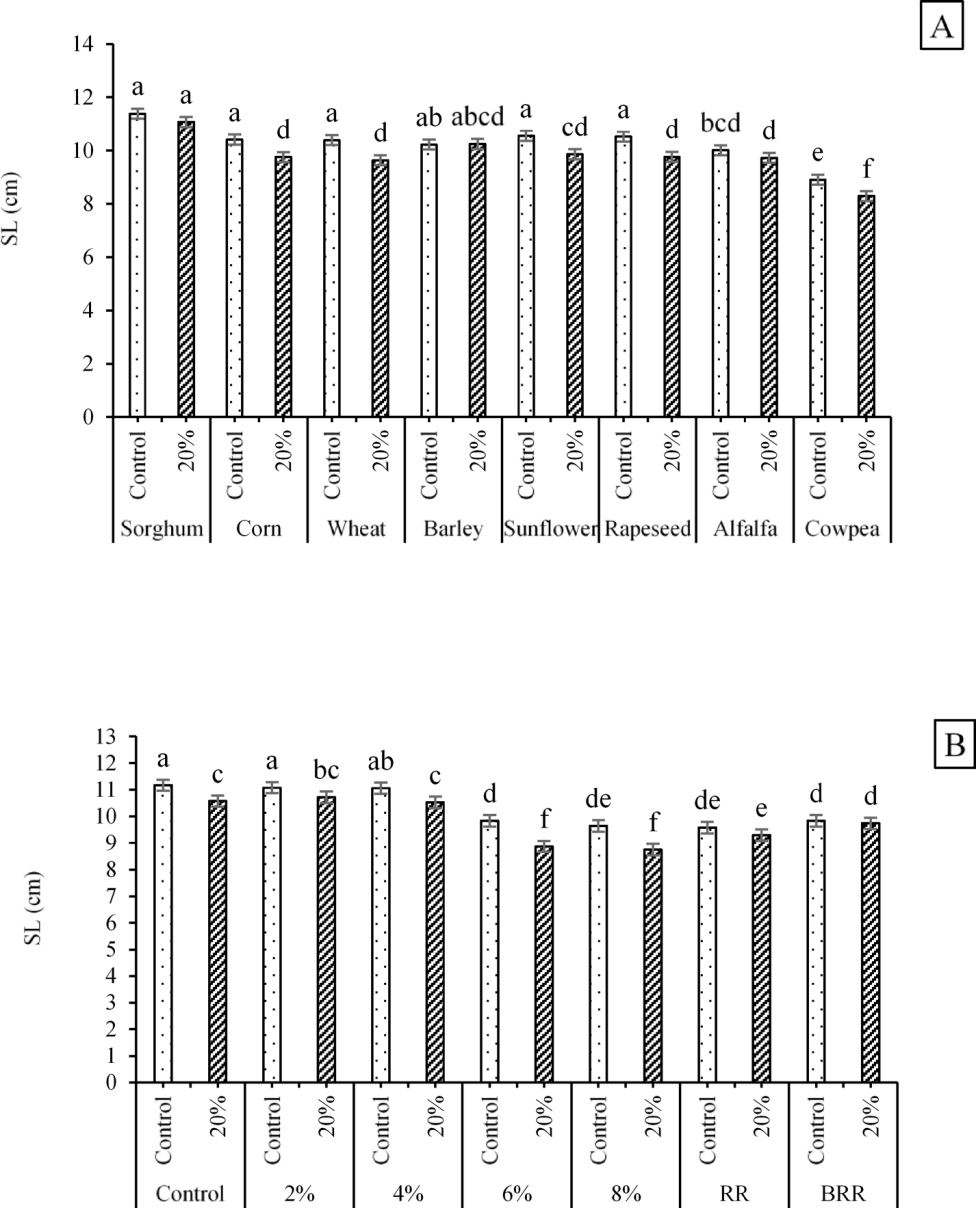
Effects of **A** ST × PEG and **B** E/R × PEG interactions on the SL trait Means sharing the same superscripts are not significantly different from each other at *p* < 0.05.

Since the SL trait was significantly influenced by both ST × PEG and E/R × PEG interactions, the corresponding results were evaluated (Fig. 2A and B). The ST × PEG interaction showed that the maximum SL value (11.39 cm) was recorded for the interaction of sorghum seeds × no PEG application. In contrast, the minimum value (8.29CM) belonged to cowpea seeds × 20% PEG interaction (Fig. 2A). Likewise, the E/R × PEG interaction indicated that the highest SL value (11.17 cm) occurred in the control × no PEG treatment, while the lowest value (8.75 cm) was observed in the 8% extract × 20% PEG treatment (Fig. 2B).

**Fig. 3:**
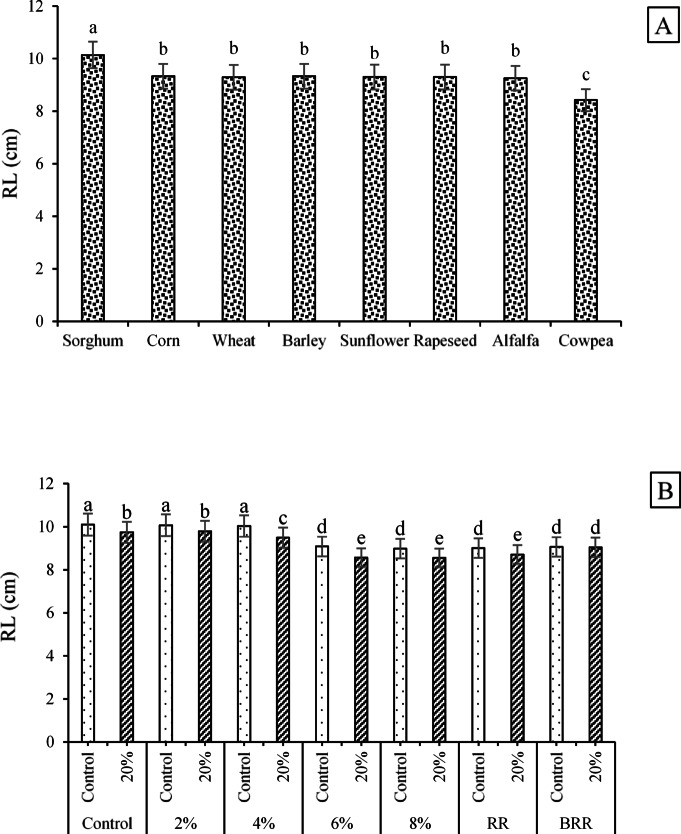
Variation in RL across **A** different seed types and **B** the interaction effects of E/R × PEG. Means sharing the same superscripts are not significantly different from each other at *p* < 0.05.

Regarding the effects of seed type on the RL trait, sorghum seeds exhibited the greatest root length (10.14 cm), whereas cowpea seeds produced the shortest roots (8.42 cm). Overall, sorghum roots were 12–18% longer than those of corn, wheat, barley, sunflower, rapeseed, alfalfa, and cowpea (Fig. 3A). For the E/R × PEG interaction, the longest roots (10.10, 10.07, and 10.03 cm) were obtained from the combinations of control × no PEG, 2% extract × no PEG, and 4% extract × no PEG, respectively. In contrast, the shortest roots (8.55, 8.56, and 8.71 cm) were observed for the combinations of 6% extract × 20% PEG, 8% extract × 20% PEG, and RR × 20% PEG, respectively (Fig. 3B).

**Fig. 4:**
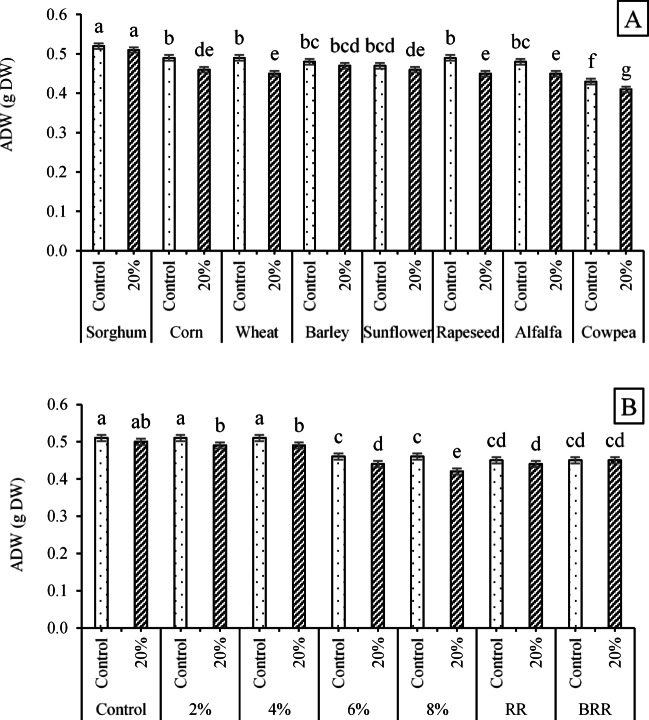
The interaction effects of **a** ST × PEG and **b** E/R × PEG on the ADW trait. Means sharing the same superscripts are not significantly different from each other at *p* < 0.05.

ADW-related mean squares (Fig. 4A) revealed that the maximum values (0.52 and 0.51 g) were recorded for the interactions of sorghum seeds × no PEG application and sorghum seeds × 20% PEG, which had significant differences compared to the obtained values in other interactions. The minimum ADW was weighted for the interaction of cowpea seeds × 20% PEG with an equivalent value of 0.41 g. In addition, considering the effects of E/R × PEG on ADW (Fig. 4B), the highest value (0.51 g dry weight) was jointly observed for the interactions of control × control (no PEG application), 2% extract × no PEG application, and 4% extract × no PEG application. The lowest ADW value (0.42 g dry weight) was also recorded for the interaction of 8% extract × 20% PEG (Fig. 4B), which had a significant decrease compared to the aerial dry weight under other interactions.

**Fig. 5:**
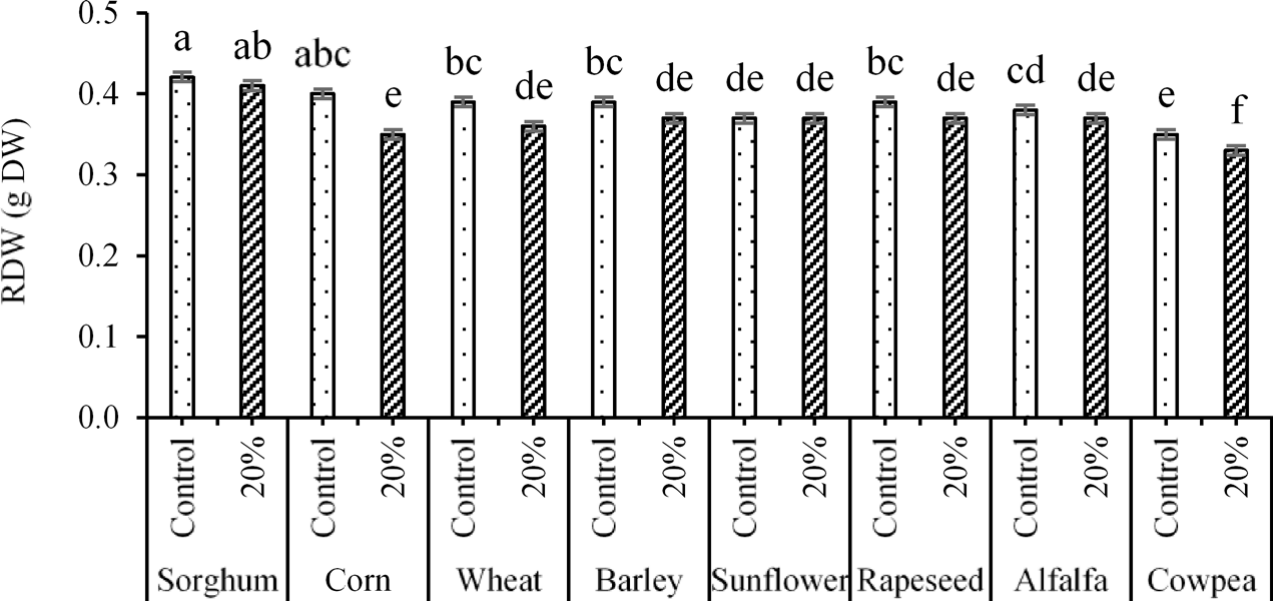
The interaction effects of ST × PEG on the RDW trait. Means sharing the same superscripts are not significantly different from each other at *p* < 0.05.

Furthermore, the notable effects of the ST × PEG interaction on RDW indicated that the highest weight (0.42 g DW) was obtained for the interaction of sorghum seeds × control (no PEG application), which had no significant increase compared to the dry weight obtained from the interactions of sorghum seeds × 20% PEG (0.41 g) and corn seeds × no PEG application (0.4 g). On the other hand, the least significant value (0.33 g DW) was obtained for the interaction of cowpea seeds × 20% PEG (Fig. 5).

#### Biochemical indices

##### Chl a, Chl b, Chl T, and CAR contents

Outputs (Table 6) showed that Chl α and Chl T were affected by ST, E/R, and PEG treatments at *p* < *0.01*. The content of Chl b was also affected by ST and E/R (*p* < *0.01*). On the other hand, ST and PEG treatments were significant at *p* < 0.01. Also, the CAR contents were significantly affected by interactions ST × E/R (*p* < *0.01*) and ST × PEG (*p* < *0.05*). However, E/R treatment and the interactions of E/R × PEG and ST × E/R × PEG did not cause significant differences in the CAR content.

**Table 6:** Analysis of variance (mean squares) of biochemical indices in petri culture conditions.

Sources	df	Chl a	Chl b	Chl T	CAR	LPC	SC	CAT	SOD	APX
Seed type (ST)	7	0.02**	0.004**	0.03**	0.0002**	4.42**	102.43**	79.59**	60.21**	39.99**
Extracts/Residues (E/R)	6	0.005*	0.002 ns	0.01**	0.00004 ns	4.00**	133.3**	106.91**	57.19**	49.73**
Polyethylene glycol (PEG)	1	0.07**	0.1**	0.35**	0.002**	1.62**	121.96**	135.05**	56.39**	57.19**
ST × E/R	42	0.001 ns	0.001 ns	0.001 ns	0.0002**	0.08ns	2.83*	2.38ns	0.83ns	1.47ns
ST × PEG	7	0.001 ns	0.001 ns	0.0004 ns	0.00007*	0.05ns	2.39ns	1.53ns	1.72ns	2.66*
E/R × PEG	6	0.0003 ns	0.00 ns	0.001 ns	0.00001 ns	0.17ns	4.86*	5.98**	2.16*	3.72**
ST × E/R × PEG	42	0.0004 ns	0.0004 ns	0.001 ns	0.00001 ns	0.09ns	1.48ns	1.70ns	0.86ns	1.04ns
Error (E)	224	0.002	0.001	0.001	0.00003	0.13	1.62	1.82	0.86	1.17
CV (%)		13.37	17.57	6.28	11.2	7.94	5.27	6.81	6.34	6.02

ns: non-significant; ** and * significant at *p* < 0.01 and *p* < 0.05, respectively.

Mean comparisons of the effects of the ST treatment on Chl a content (Table 7) showed that the highest value (0.33 mg g-1 FW) was obtained for sorghum seeds, which did not differ significantly from the Chl a content observed in sunflower seeds. In contrast, the lowest Chl a content (0.26 mg g^-1^ FW) was recorded in cowpea seeds, indicating a significant reduction compared with the chlorophyll a content measured in the other plant seeds. Additionally, the E/R treatment results revealed that the highest Chl a content (0.33 mg g^-1^ FW) occurred in the control treatment (no application of extract or residue); however, the lowest Chl a content (0.30 mg g^-1^ FW) was jointly observed under the 6% extract, 8% extract, and RR treatments. Furthermore, the effects of the ST treatment on Chl T content indicated that the highest and lowest values (0.54 and 0.44 mg g^-1^ FW, respectively) were obtained in sorghum and cowpea seeds, respectively. Under the E/R treatment, the highest and lowest Chl T contents (0.51 and 0.47 mg g^-1^ FW, respectively) were observed in the control and 8% extract treatments.

**Table 7:** Mean comparisons (mean squares) of ST and E/R treatments on the pigments in petri culture conditions.

Sources	Chl a (mg g^-1^ FW)	Chl T (mg g^-1^ FW)	LPC (mg g^-1^ FW)
ST treatment			
Sorghum	0.33 a	0.54 a	3.96 c
Corn	0.31b	0.49 b	4.46 b
Wheat	0.31 b	0.49 b	4.4 b
Barley	0.31 b	0.50 b	4.5 b
Sunflower	0.32 ab	0.49 b	4.43 b
Rapeseed	0.31 b	0.50	4.47 b
Alfalfa	0.31 b	0.49	4.47 b
Cowpea	0.26 c	0.44	5.16 a
LSD	0.017	0.013	0.15
E/R treatment			
Control	0.33 a	0.51 a	4.16 c
2%	0.32 ab	0.50 ab	4.18 c
4%	0.31 b	0.50 ab	4.18 c
6%	0.30 b	0.48 cd	4.69 b
8%	0.30 b	0.47 d	4.67 b
RR	0.30 b	0.48 cd	4.65 b
BRR	0.31 b	0.49 bc	4.81 a
LSD	0.017	0.01	0.14

Means sharing the same superscripts are not significantly different from each other at *p* < 0.05.

**Fig. 6:**
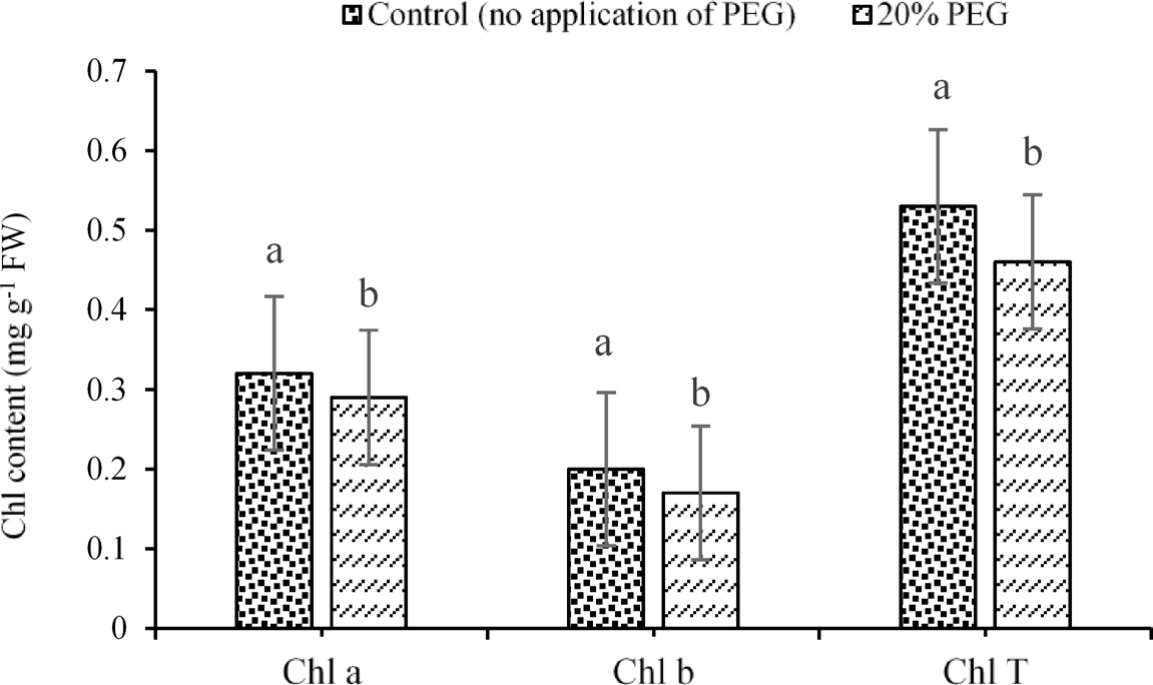
Effects of PEG on the traits of Chl a, Chl b, and Chl T. Means sharing the same superscripts are not significantly different from each other at *p* < 0.05.

Apart from the above results, the data showed that Chl a and Chl T contents decreased by 10.34% and 15.22%, respectively, under the application of 20% PEG. Overall, Chl a content was estimated to be 0.32 and 0.29 mg g^-1^ FW under the control and 20% PEG treatments, respectively. These values were obtained for Chl T equal to 0.53 and 0.46 mg g^-1^ FW (Fig. 6).

**Fig. 7:**
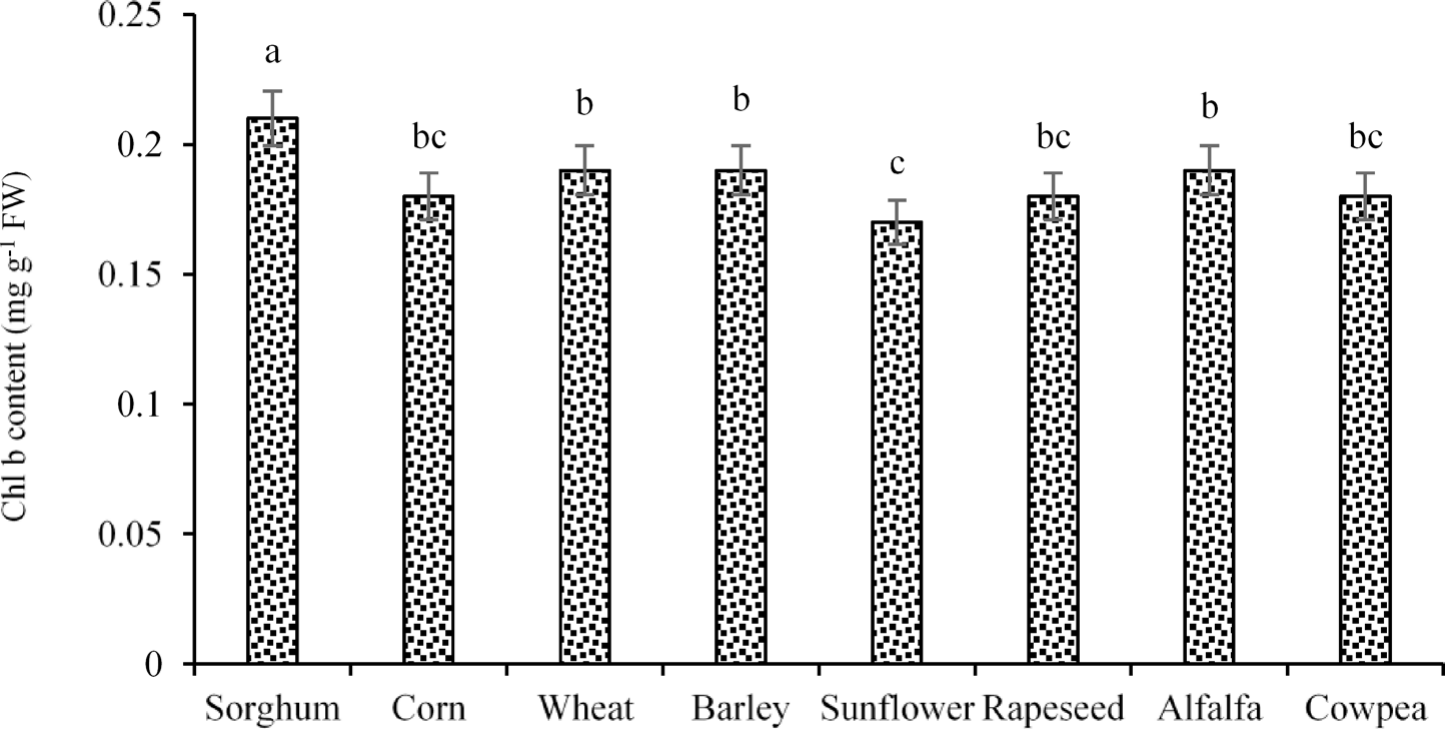
Changes in the Chl b under the application of **a** ST and **b** E/R treatments. Means sharing the same superscripts are not significantly different from each other at *p* < 0.05.

Regarding the effects of the experimental treatments (i.e., ST and E/R) on Chl b content, the results indicated that the highest and lowest Chl b contents (0.21 and 0.17 mg g-1 FW, respectively) were recorded for sorghum and sunflower seeds (Fig. 7). In addition, application of 20% PEG resulted in a significant 15% reduction in Chl b content compared with the control treatment (no PEG), such that Chl b contents under the control and 20% PEG treatments were estimated at 0.20 and 0.17 mg g-1 FW, respectively (Fig. 6; Chl b data).

**Fig. 8:**
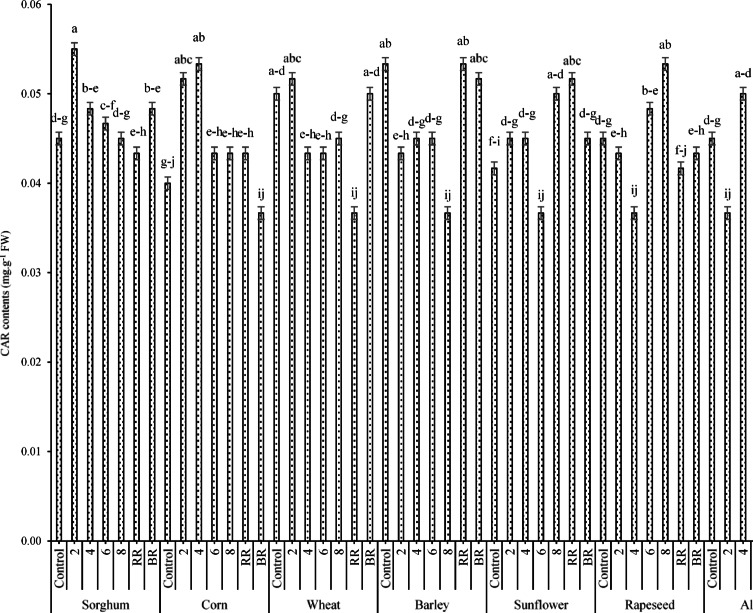
Effects of ST × E/B interaction on the CAR content. Means sharing the same superscripts are not significantly different from each other at *p* < 0.05.

Figure 8 illustrates the changes in CARs under the two-way interaction of ST × E/R. Based on the results, the highest CAR content (0.055 mg g^-1^ FW) was obtained for the interaction of sorghum × 2% extract, and the lowest content was observed for the interaction of cowpea × BR (0.035 mg g^-1^ FW).

**Fig. 9:**
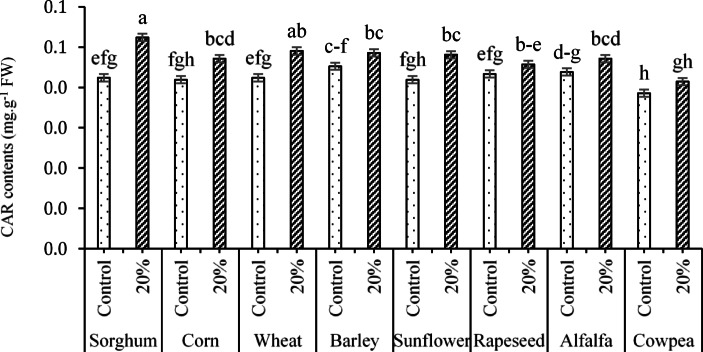
Effects of ST × E/B interaction on the CAR content. Means sharing the same superscripts are not significantly different from each other at *p* < 0.05.

Since CARs also affected by the two-way interaction of ST × PEG, the highest and lowest values (0.052 and 0.039 mg g^-1^ FW, respectively) were obtained for the interactions of control × PEG and BRR × no PEG (Fig. 9).

#### Leaf proline content (LPC)

ANOVA results (see Table 6) demonstrated that LPC was affected by all main treatments of ST, E/R, and PEG application at p < 0.01. However, none of their two-way and three-way interactions caused a significant difference in the content of the aforementioned attribute.

**Fig. 10:**
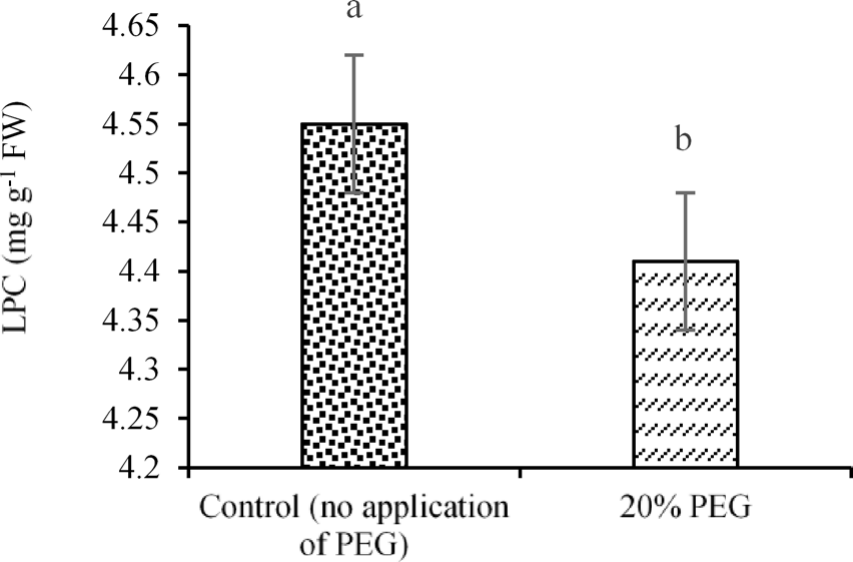
Effects of ST × E/B interaction on the CAR content. Means sharing the different superscripts are significantly different from each other at *p* < 0.05.

According to mean comparisons (Table 7), cowpea seedlings exhibited the highest LPC at 5.16 mg g^-1^ FW, while sorghum seedlings had the lowest LPC (3.96 mg g^-1^ FW). Regarding the effects of E/R treatment, the highest LPC (4.81 mg g^-1^ FW) was observed in the BRR treatment. The lowest value (3.96 mg g^-1^ FW) was also recorded for the control treatment. Furthermore, the application of 20% PEG reduced LPC slightly (4.41 mg g⁻^1^ FW), representing a 3.08% decrease compared to the control (no PEG) treatment with an average value of 4.55 mg g^-1^ FW (Fig. 10).

#### Soluble carbohydrates (SC)

Concerning the effects of treatments on non-enzymatic antioxidants, significant effects of main treatments (ST, E/R, and PEG application) were estimated at p < 0.01, along with significant effects of ST × E/R and E/R × PEG at p < 0.05 on the SC trait (see Table 6).

**Fig. 11:**
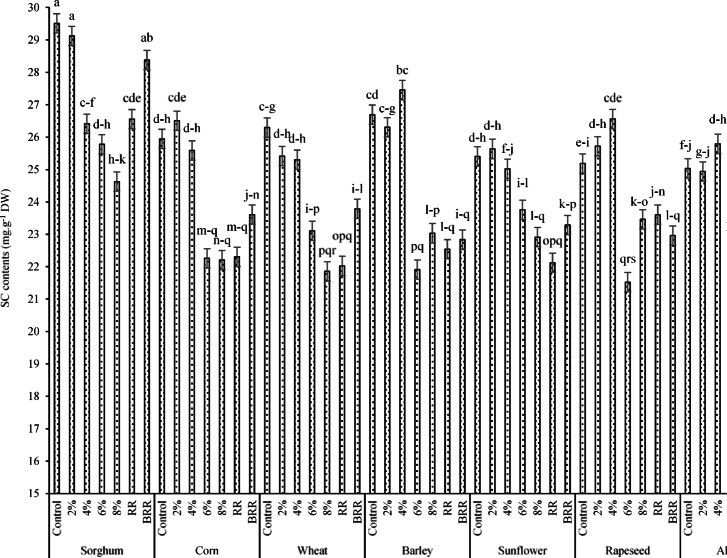
Effects of ST × E/B interaction on the SC content. Means sharing the different superscripts are significantly different from each other at *p* < 0.05.

According to the data (Fig. 11), the mean squares confirmed that the highest SC contents (29.51mg g^-1^ DW) was obtained for interactions of sorghum × control (no PEG application), which showed a non-significant difference between them. The lowest SC contents were also evaluated for interactions of cowpea × 8% extract (19.57 mg g^-1^ DW) and cowpea × RR (19.66 mg g^-1^ DW).

**Fig. 12:**
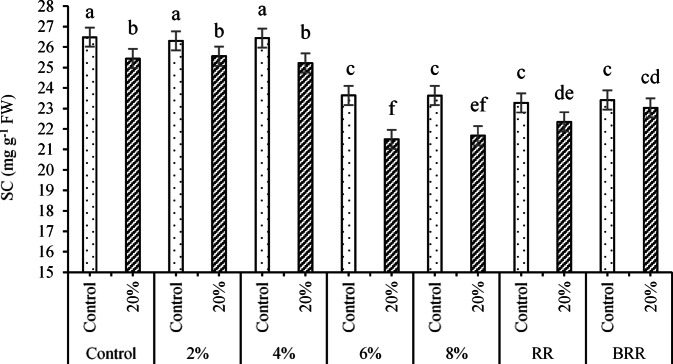
Effects of E/B × PEG interaction on the SC content. Means sharing the different superscripts are significantly different from each other at *p* < 0.05.

The results of the data related to the effects of E/B × PEG interaction (Fig. 12) also indicated that the highest and lowest SC contents (equal to 26.48 and 21.49 mg g^-1^ DW, respectively) were acquired for interactions of control (no extract application) × no PEG application and 6% extract × 20% PEG.

#### Catalase (CAT), Superoxide dismutase (SOD), and Ascorbate peroxidase (APX) activities

**Fig. 13:**
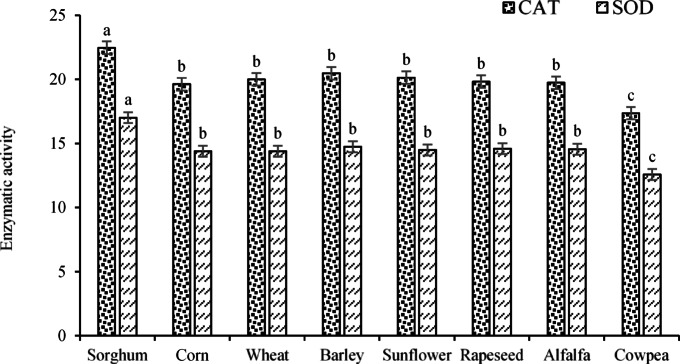
Effects of ST treatment on the enzymatic activities of CAT and SOD. Means sharing the different superscripts are significantly different from each other at *p* < 0.05.

Based on the ANOVA results, the enzymatic activity of CAT was affected by ST, E/R, and PEG treatments, as well as the interaction of E/R × PEG (*p* < 0.01). In addition, SOD was significantly affected by ST, E/R, and PEG parameters at *p* < 0.01 and by the interaction of E/R × PEG at *p* < 0.05. In the meantime, ST, E/R, and PEG treatments, together with the interaction of E/R × PEG at *p* < 0.01 and the interaction of ST × PEG at *p* < 0.05, had significant effects on the enzymatic activity of APX. Mean comparisons related to the ST treatment on the enzymatic activity of CAT (Fig. 13) illustrated that the highest and lowest values (22.46 and 17.36 mM H_2_O_2_ mg^-1^ pr min^-1^) were calculated for sorghum and cowpea seedlings, respectively. The results of ST treatment on SOD activity were also similar, with the highest and lowest values recorded for sorghum and cowpea seedlings (17.02 and 12.57 U mg^-1^ pr), respectively.

**Fig. 14:**
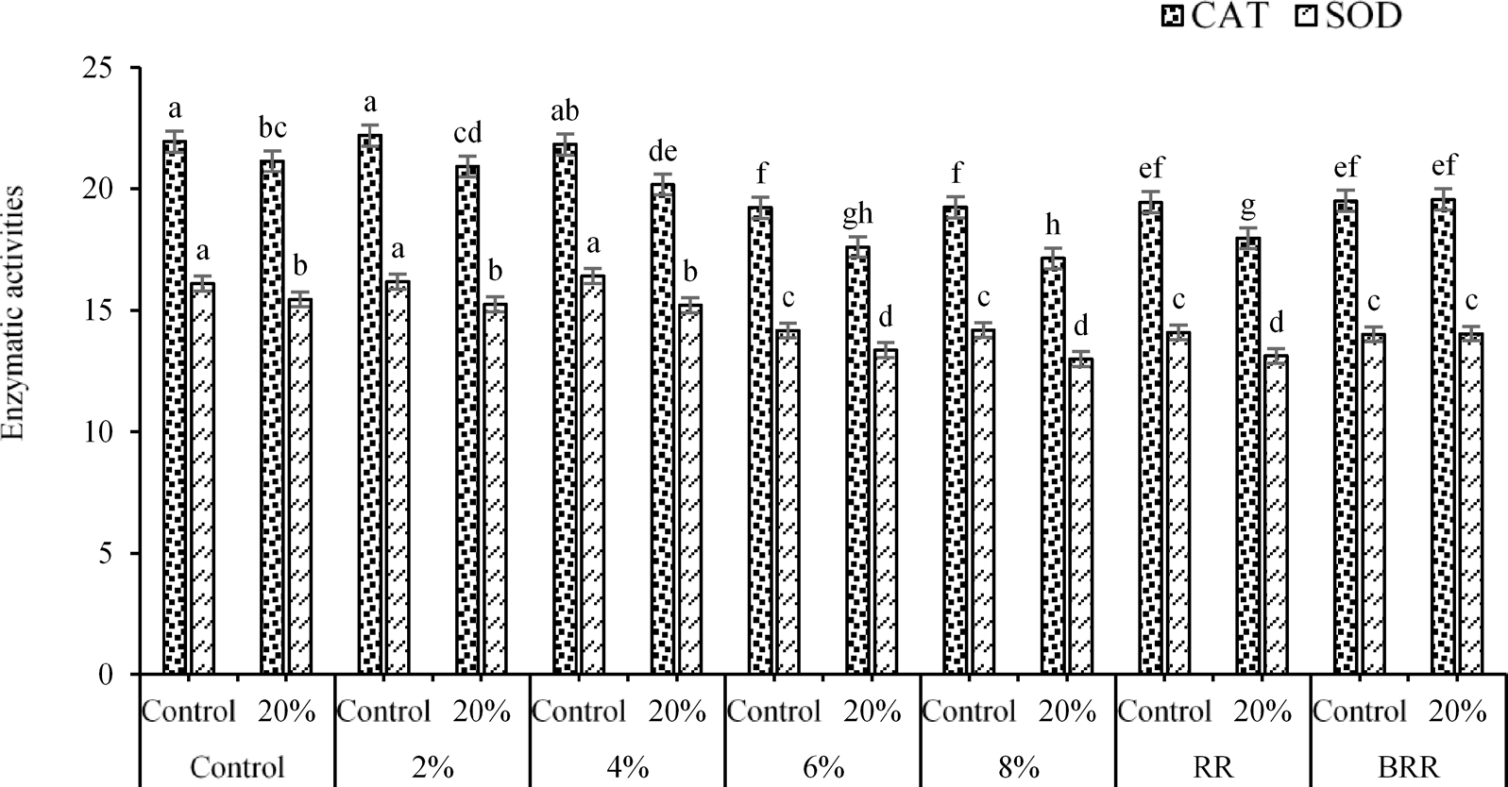
Effects of the interaction of E/R × PEG on the enzymatic activities of CAT and SOD. Means sharing the different superscripts are significantly different from each other at *p* < 0.05.

The results of E/B × PEG interaction on enzymatic activities (Fig. 14) showed that the highest and lowest CAT activity (with averages of 22.19 and 17.13 mM H_2_O_2_ mg^-1^ pr min^-1^) were calculated under 2% extract × control (no PEG ) and 8% extract × 20% PEG interactions, respectively. The highest and lowest enzymatic activity of SOD (with average values of 16.41 and 12.98 U mg^-1^ pr) were assessed for interactions of 4% extract × control (no PEG application) and 8% extract × 20% PEG. Furthermore, the results of the E/B × PEG interaction on enzymatic activities showed that the highest and lowest CAT activity (with averages of 22.19 and 17.13 mM H_2_O_2_ mg^-1^ pr min^-1^) were calculated under 2% extract × control (no PEG application) and 8% extract × 20% PEG interactions, respectively. The highest and lowest activity of the SOD enzyme (with average values of 16.41 and 12.98 U mg^-1^ pr) were assessed for interactions of 4% extract × control (no PEG application) and 8% extract × 20% PEG.

**Fig. 15:**
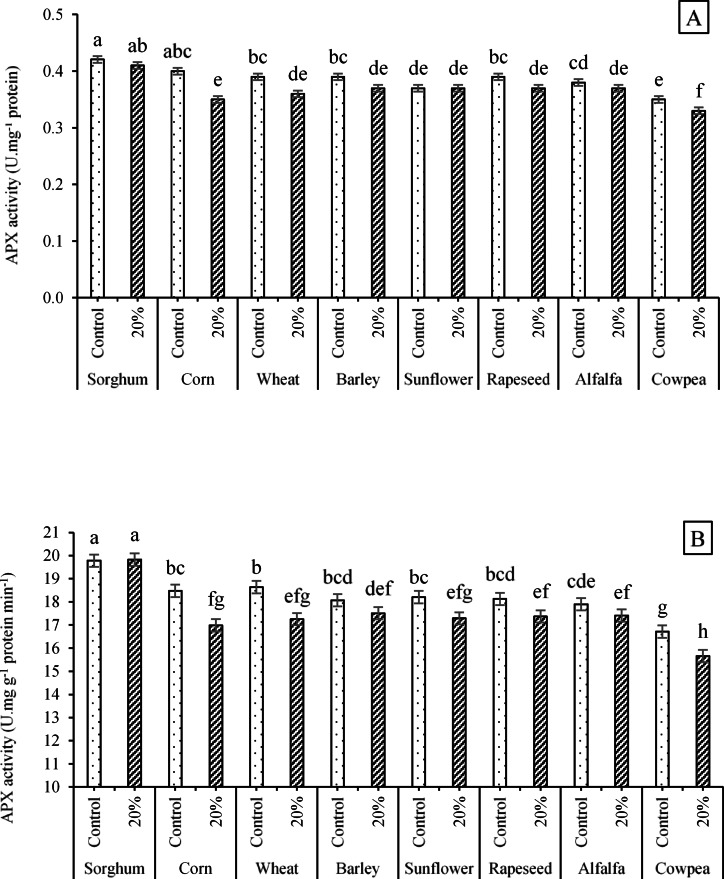
Effects of the interactions **A** ST × PEG and **B** E/R × PEG on the enzymatic activities of APX. Means sharing the different superscripts are significantly different from each other at *p* < 0.05.

In addition to the above, it was observed that the enzymatic activity of APX was influenced by the interactions of ST × PEG and E/R × PEG (Table 6). According to the mean comparisons, the interaction of ST × PEG resulted in significant changes in the APX activity. The highest values (19.83 and 19.78 U mg^-1^ protein min^-1^) were achieved for interactions of sorghum × 20% PEG and sorghum × no PEG application, which were not significantly different from each other. The lowest values (equal to 15.66 and 16.71 U mg^-1^ protein min^-1^) were estimated for interactions of cowpea × 20% PEG and cowpea × no PEG application, respectively (Fig. 15A). Data also confirmed a significant increase in APX activity by the interaction of E/R × PEG, so that the highest value (19.41 U mg^-1^ protein min^-1^) was observed doe the interaction of control (no E/R application) × no PEG application, which had no significantly different with values of APX under the interactions of 2% extract × no PEG application (19.17 U mg^-1^ protein min^-1^) and 4% extract × no PEG application (19.37 U mg^-1^ protein min^-1^). The least APX activity (15.98 U mg^-1^ protein min^-1^) was also evaluated for the interaction of 8% extract × 20% PEG (Fig. 15B).

#### Greenhouse (pot) experiments

In this experiment, the most sensitive plants in the previous step were identified and excluded from further research. Hence, greenhouse experiments were conducted with more tolerant plants including sorghum, corn, wheat, barley, sunflower, and rapeseed.

#### Pigments (Chl a, Chl b, Chl T, and CAR contents)

The results, as shown in Table 8, indicated that Chl a, Chl b, and Chl T exhibited significant changes under the application of the main treatments of plant type (PT), root residues (RR), and application conditions (AC) of the root residues *p* < 0.01. However, their interactions had no notable changes in the content of the aforementioned pigments. In addition, all PT, RR, and AC treatments, as well as their interactions (i.e., PT × RR, PT × AC, RR × AC, and PT × RR × AC) created significant differences in CAR contents at *p* < 0.01 (Table 8).

**Table 8:** Analysis of variance (mean squares) of biochemical indices in greenhouse (pot) conditions.

Sources	df	Chl a	Chl b	Chl T	CAR	LPC	SC	TPC	CAT	SOD
Plant type (PT)	5	0.02**	0.01**	0.05**	0.0004**	0.75**	277.46**	1.67**	80.93**	5.89**
Root residues (RR)	1	0.07**	0.02**	0.18**	0.0014**	3.7**	816.05**	5.78**	299.01**	252.31**
Application conditions (AC)	3	0.08**	0.01**	0.14**	0.001**	2.56**	511.45**	2.71**	241.41**	135.19**
PT × RR	5	0.0001 ns	0.0004 ns	0.0004 ns	0.0001**	0.04ns	42.55ns	0.19ns	13.01ns	5.37ns
PT × AC	15	0.002 ns	0.001 ns	0.001 ns	0.0001**	0.06ns	57.86*	0.11ns	8.65ns	6.39ns
RR × AC	3	0.001 ns	0.0001 ns	0.001 ns	0.0002**	0.02ns	34.79ns	0.04ns	16.01ns	5.88ns
PT × RR × AC	15	0.0004 ns	0.001 ns	0.001 ns	0.0001**	0.05ns	40.47*	0.16ns	4.23ns	3.81ns
Error (E)	96	0.001	0.0007	0.001	0.00003	0.08	22.83	0.2	7.41	5.04
CV (%)		6.75	13.62	4.80	9.16	7.81	11.7	13.66	11.33	13.34

ns: non-significant; ** and * significant at *p* < 0.01 and *p* < 0.05, respectively.

As can be seen (Table 9), the highest and lowest values of the Chl a content in greenhouse conditions (0.55 and 0.46 mg g^-1^ FW), chl b (0.22 and 0.18 mg g^-1^ FW), and Chl T (0.77 and 0.64 mg g^-1^ FW) were obtained for sorghum and rapeseed plants. Also, the data demonstrated a significant decrease in the content of Chl α, Chl b, and Chl T under the application of RR. According to the results, applying RR treatment decreased Chl α, Chl b, and Chl T equal to 7.69%, 14.29%, and 9.59% compared to the control treatment. Furthermore, the AC treatment also influenced photosynthetic pigments in this study. Based on the results, the highest and lowest contents of Chl a (0.56 and 0.45 mg g^-1^ FW), Chl b (0.21 and 0.17 mg g^-1^ FW), and Chl T (0.77 and 0.62 mg g^-1^ FW) occurred under the T3 (three months before planting) and T1 (one month before planting ) treatments (Table 9).

**Table 9:** Mean comparisons (mean squares) of ST and E/R treatments on the pigments in petri culture conditions.

Sources	Chl a (mg g^-1^ FW)	Chl b (mg g^-1^ FW)	Chl T (mg g^-1^ FW)	LPC (mg g^-1^ FW)	TPC (mg GAE g^-1^)	CAT (mM H_2_O_2_ mg^-1^ pr min^-1^)	SOD (U mg^-1^ pr min^-1^)
PT treatment							
Sorghum	0.55 a	0.22 a	0.77 a	3.3 d	3.59 a	26.92 a	19.63 a
Corn	0.52 b	0.2 b	0.72 b	3.54 bc	2.99 cd	21.94 c	17.66 bc
Wheat	0.51 b	0.2 b	0.71 b	3.5 c	2.89 d	23.44 bc	16.81 cd
Barley	0.48 b	0.19 bc	0.67 c	3.74 a	3.24 bc	23.52 b	16.59 cd
Sunflower	0.48 b	0.19 bc	0.67 c	3.69 ab	3.43 ab	22.85 bc	15.48 d
Rapeseed	0.46 b	0.18 c	0.64 d	3.76 a	3.37 ab	25.49 a	18.35 ab
LSD	0.02	0.01	0.02	0.16	0.25	1.56	1.33
RR treatment							
Control	0.52	0.21 a	0.73 a	3.43 b	3.05 b	25.47 a	18.74 a
RR	0.48	0.18 b	0.66 b	3.75 a	3.45 a	22.59 b	16.09 b
LSD	0.01	0.01	0.01	0.09	0.15	0.9	0.77
Application conditions (AC)							
At the time of planting (T0)	0.51 b	0.19 b	0.70 b	3.52 b	3.61 a	27.16 a	17.8 a
One month before planting (T1)	0.45 d	0.17 c	0.62 d	3.92 a	3.3 b	24.82 b	17.61 b
Two months before planting (T2)	0.49 c	0.19 b	0.68 c	3.64 b	3.14 bc	23.08 c	17.21 b
Three months before planting (T3)	0.56 a	0.21 a	0.77 a	3.28 c	2.96 c	21.06 d	15.07 c
LSD	0.02	0.01	0.01	0.13	0.21	1.27	1.08

Means sharing the same superscripts are not significantly different from each other at *p* < 0.05.

**Fig. 16:**
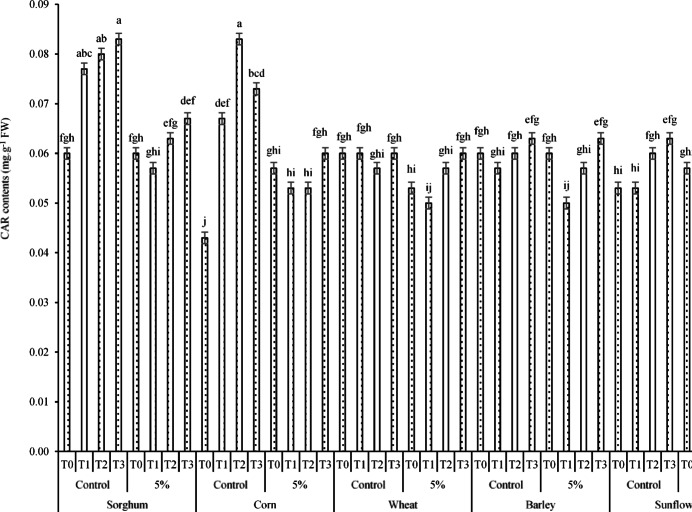
Changes in CARs under the effects three-way interaction of PT × RR × AC. Means sharing the different superscripts are significantly different from each other at *p* < 0.05.

According to the effects of the three-way interaction of PT × RR × AC on CAR content (Fig. 16), the results indicated that the highest CAR content (0.083 mg g^-1^ FW) was jointly observed under the interactions of sorghum × no RR (control) × T3 and corn × no RR × T2. However, no significant difference was detected between this value and the CAR contents obtained from the interactions of sorghum × no RR × T1 (0.077 mg g^-1^ FW) and sorghum × no RR × T2 (0.080 mg g^-1^ FW). In contrast, the lowest CAR content (0.043 mg g^-1^ FW) was estimated under the interaction of corn × no RR × T0 (Fig. 16).

#### Soluble carbohydrates (SC), Leaf proline content (LPC), and total phenolic content (TPC)

**Fig. 17:**
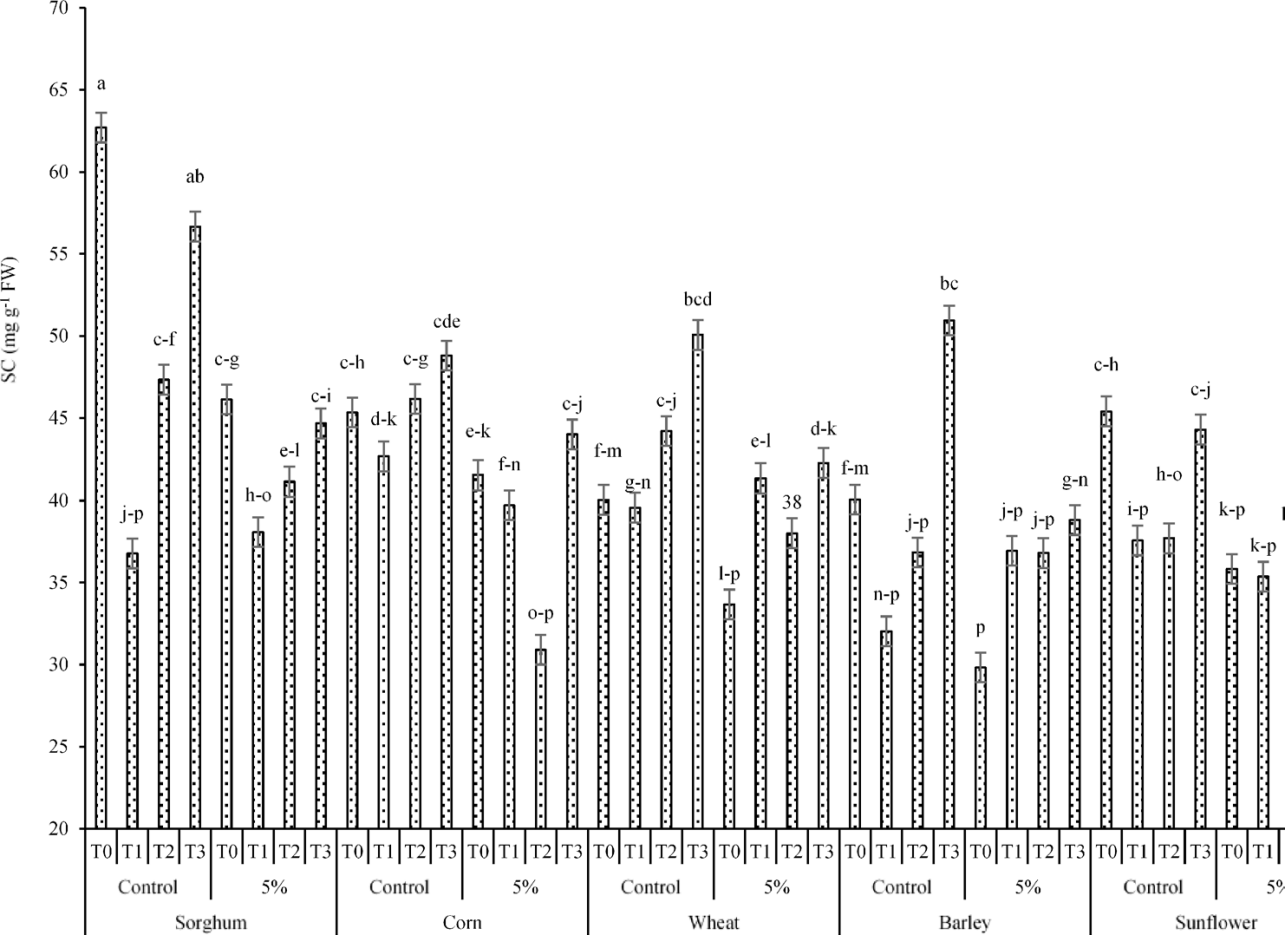
Effects of the interaction of PT × RR × AC on SC t. Means sharing the different superscripts are significantly different from each other at *p* < 0.05.

Results of the ANOVA (Table 8) demonstrated that SC was affected by the by the main treatments of PT, RR, and AC (*p* < *0.01*) and interactions of PT × AC and PT × RR × AC (*p* < *0.05*). In addition, LPC and TPC were affected by the PT, RR, and AC treatments at *p* < 0.01. Regarding the effects of the three-way interaction of PT × RR × AC on SC (Fig. 17), the resulting data confirmed that the highest and lowest content of the above trait (equal to 62.7 and 29.83 mg g^-1^ DW) occurred under the interactions sorghum × control (no RR application) × T0 and barley × 5%RR × T0, respectively.

According to the effects of PT treatment (Table 9), mean squares indicated that the maximum LPC (equal to 3.76 mg g^-1^ FW) was assessed for rapeseed plants. The obtained data also confirmed that LPC in rapeseed plants was enhanced by 12.23%, 5.85%, 6.91%, 0.53%, and 1.86% compared to sorghum, corn, wheat, barley, and sunflower, respectively. In addition, the treatment RR caused a significant increase of 9.33% in the mentioned trait compared to the control treatment (with an average value of 3.43 mg g^-1^ FW). Furthermore, the minimum and maximum proline concentrations under AC treatment (3.28 and 3.92 mg g^-1^ FW) were evaluated for T1 and T3 treatments.

As shown in Table 9, TPC was affected by the main treatments in the present study. Mean comparisons of the PT treatment showed that sorghum plants had the highest TPC (3.59 mg GAE g^-1^), while the lowest TPC (2.89 mg GAE g^-1^) was extracted from cultivated wheat plants. Mixing RR with potting soil also resulted in a significant 13.11% increase in TPC in the tested plants. Based on the data, TPC was evaluated under RR treatment at an average of 3.45 mg GAE g^-1^. In comparison, its content was estimated to be 3.05 mg GAE g^-1^ under control treatment (no RR application). Apart from the above, investigating the effects of AC treatment also showed that mixing RR with the pot soil at the time of planting (T0) significantly led to increases of 9.39%, 14.97%, and 21.96% in TPC compared to T1, T2, and T3 treatments (Table 9).

#### CAT and SOD activity

The CAT and SOD activities were significantly affected by PT, RR, and AC treatments at p < 0.01. However, despite the significant effects of the main treatments, non-significant changes were observed for both of the mentioned traits under their interactions (Table 8).

Among examined plants (Table 9), sorghum and rapeseed plants had the highest enzymatic activities of CAT (26.92 and 25.42 mM H_2_O_2_ mg^-1^ pr min^-1^) and SOD (means 19.63 and 18.35 U mg^-1^ pr min^-1^). In contrast, the lowest CAT enzymatic activity (21.94 mM H_2_O_2_ mg^-1^ pr min^-1^) was measured in corn plants, while the lowest SOD value (15.48 U mg-1 pr min^-1^) was estimated in sunflower plants. The mean comparison data also indicated that the application of RR treatment significantly reduced CAT and SOD enzymatic activities by 12.75% and 16.47%, respectively, compared to the control treatment (no RR application). In addition, mixing sorghum root residues with potting soil (i.e., AC treatment) also had a meaningful effect on the content of both enzymatic activities. Based on the outputs, the highest activity of CAT (27.16 mM H_2_O_2_ mg^-1^ pr min^-1^) was acquired for the T0 application, which showed a significant increase of 9.43, 17.68, and 28.96% compared to T1, T2, and T3 treatments. Data analysis also revealed that the highest enzymatic activity for SOD (17.8 U mg^-1^ pr) was observed for T0 treatment (Table 9).

## Discussion

The findings of this study highlight the significant allelopathic and autotoxic effects of sorghum extracts, as well as the impacts of PEG-induced osmotic stress on germination-related indices, seedling vigor, and biomass accumulation in eight plant species. In general, the results provide valuable insights into the interactions between allelochemical phytotoxicity and drought-related stress responses, demonstrating that the combined application of sorghum extracts and PEG intensifies inhibitory effects on germination indices and early growth traits. The clear dose-dependent response (particularly the strong inhibition observed at 8% extract) confirms the concentration threshold at which sorghum allelochemicals exert critical phytotoxicity. The combined application of 6–8% extracts with 20% PEG caused severe reductions in GP, radicle and plumule elongation, and ADW (biomass production). Sorghum displayed exceptional resilience under the combined stresses of 8% extract and 20% PEG, consistently outperforming all other evaluated species in terms of germination, seedling growth, and physiological responses. These observations supported earlier findings^33–35^ and indicated that sorghum’s intrinsic stress-resistance mechanisms remain effective even under combined pressures of allelopathic and osmotic stress. The findings of the present study further demonstrated that plant species differed in their responses to environmental stress in terms of photosynthetic pigments. Notably, sorghum exhibited the highest chlorophyll and carotenoid contents among the tested species. In this context, Norouzi and Akbari^36^ reported that biochemical responses, particularly chlorophyll and carotenoid levels, vary significantly among plant species and cultivars. Reductions in chlorophyll content may result from chlorophyll degradation, increased oxidative stress, and disruptions in the activity of chlorophyll-associated enzymes, whereas more tolerant genotypes possess a greater capacity for chlorophyll synthesis and retention. Likewise, positive and negative variations in carotenoid content have been linked to their functional roles in protecting chlorophyll, enhancing plant tolerance to adverse environmental conditions, and supporting other related protective mechanisms. Corn also displayed strong tolerance, likely due to efficient detoxification pathways, as Sytykiewicz^37^ demonstrated the presence of glutathione-S-transferase (GST) isoforms capable of rapidly metabolizing allelochemicals. In contrast, legumes showed high sensitivity, highlighting the importance of species selection in allelopathic environments, consistent with the view of Robin et al.^38^ that species performance depends on root architecture and inherent stress-response pathways.

The phytotoxicity observed at higher extract concentrations aligns with established mechanisms of sorghum allelopathy. Sorgoleone and phenolic acids^18,39,40^ are known to disrupt mitochondrial respiration, inhibit ATP synthesis^41,42^, and interfere with hormone regulation and membrane integrity^39^. By integrating extract-induced toxicity with PEG-driven osmotic stress, the present study illustrates how biochemical inhibition and water-deficit signals interact to intensify physiological disruption. The dose-dependent decline in germination velocity and early seedling growth further supports this synergistic effect.

Stress-induced decreases in photosynthetic pigments were associated with concomitant increases in both enzymatic (e.g., CAT, SOD, APX) and non-enzymatic antioxidants, highlighting the pivotal role of oxidative stress in growth inhibition. These patterns are consistent with previous reports^43–45^ and were clearly observed in the present study. These responses, observed across most species, reflect the central role of oxidative damage in growth suppression under allelopathic and osmotic stress. Increased activities of CAT, SOD, and APX, along with enhanced proline and soluble carbohydrate levels, indicate activation of ROS-mitigating pathways^46^. These findings are consistent with the role of antioxidant systems in stress tolerance as emphasized by Foyer & Noctor^47^. In addition, the combined application of allelopathic extracts and PEG exacerbated stress responses, suggesting a synergistic effect between chemical inhibition and osmotic disruption^48^. The addition of PEG-induced osmotic stress created a novel experimental system that revealed important interactions between allelochemical and drought stress pathways. Our data show that the combination of these stressors often produced synergistic effects, particularly in species with known drought sensitivity. The strong decline in chlorophyll content under combined stresses suggests that the photosynthetic apparatus is among the primary targets of allelochemical, osmotic interactions, confirming the observations of Hussain et al.^18^. By incorporating PEG-induced osmotic stress as an additional experimental variable, the current study provides new insights into the interaction between allelopathic and drought-related stresses. These findings are in agreement with previous reports documenting PEG-induced stress responses in plants^38,44,49^. Additionally, antioxidant enzyme activities displayed a biphasic response, whereby moderate stress significantly enhanced CAT and SOD activities. In contrast, severe stress exceeded the capacity of these systems, leading to diminished antioxidant effectiveness. A similar finite ROS-scavenging capacity has been previously reported by Gill & Tuteja^50^. Sorghum, rapeseed, sunflower, and corn successfully maintained antioxidant functionality at higher stress levels, suggesting promising tolerance traits for breeding and selection programs. Overall, the study demonstrates that sorghum possesses superior tolerance to both allelochemical and osmotic stresses, consistent with reports showing its exceptional adaptive capacity^51–53^. The strong phytotoxicity of 6–8% extracts is also supported by recent studies identifying several previously undocumented phenolic compounds in sorghum root exudates^18,44^. Notably, the complete inhibition of germination in sensitive legumes at 8% extract underscores their vulnerability, likely due to thin seed coats and higher metabolic activity during early germination.

Regarding root residues, the study showed that fresh residues possess a distinct allelochemical profile enriched with persistent compounds embedded within a cellulose–hemicellulose matrix. This structure creates a slow-release system that prolongs phytotoxicity. Lignin content (14.8 mg.g^-1^) contributes to this sustained release, as lignin-bound phenolics can remain bioactive for up to eight weeks^54,55^. Burned residues behaved differently: alkaline pH (9.3) and elevated mineral levels (7.45 mg.g^-1^ K⁺) generated immediate toxicity. Although most phenolics degrade during burning, the presence of compounds such as 5-hydroxymethylfurfural, combined with ash-derived minerals, creates a distinct and rapid phytotoxic profile, explaining the root tip necrosis observed. Additionally, the greenhouse experiments further validated the initial results, demonstrating that the timing of incorporating root residue into the soil had a significant impact on plant growth and development. Hence, those that received sorghum residues three months before planting exhibited the highest chlorophyll levels and reduced stress-induced physiological responses. This can support that the delayed incorporation of residues can enhance soil health and improve subsequent crop performance. Ongoing analyses are expected to provide a deeper understanding of the synergistic effects of sorghum root residues on crop performance, offering potential improvements in sustainable residue management practices. Future field trials involving the six resilient species in soils enriched with various sorghum residues will further clarify the long-term impacts on growth and physiological responses. Our findings will be reported as soon as they are available.

## Conclusions

This study highlights the differential responses of eight plant species against sorghum-derived allelochemicals, root residues, and PEG-induced osmotic stress under controlled Petri dish and greenhouse conditions. Significant variation was observed in germination parameters, seedling vigor, photosynthetic pigments, antioxidant enzyme activities, and growth traits. Sorghum and corn consistently exhibited superior tolerance, likely due to inherent allelopathic resistance, efficient detoxification pathways such as GST-mediated metabolism in corn, and adaptive root and physiological traits. Conversely, legumes, particularly alfalfa and cowpea, were highly susceptible to combined allelochemical and drought stress, reflecting their limited antioxidant buffering and higher metabolic sensitivity during early growth. The timing and composition of root residue incorporation also critically influenced plant responses, with delayed application reducing phytotoxic pressure and supporting chlorophyll retention and growth. Collectively, these findings highlight the importance of species selection, residue management, and stress mitigation strategies for sustainable cropping systems. Future field-based studies are warranted to validate these results and inform practical recommendations for cultivating stress-resilient crops in sorghum-dominated and water-limited agroecosystems.

## Data Availability

The datasets generated, used, deployed, and analyzed during the present study are not accessible to the general public. However, they are available from the corresponding author upon adequate request.

## Abbreviations

ADW: Aerial dry weight
APX: Ascorbate peroxidase
CAR: Carotenoids
CAR: Carotenoids
CAT: Catalase
Chl α: Chlorophyll α
Chl b: Chlorophyll b
Chl T: Total chlorophyll
CV: Coefficient of variation
DW: Dry weight
FW: Fresh weight
GP: Germination percentage
LPC: Leaf proline content
MTG: Mean of time germination
Pr: Protein
PT: Plant types
RDW: Root dry weight
RL: Root length
RSR: Root to shoot ratio
SC: Soluble carbohydrate
SG: Speed of germination
SL: Shoot length
SOD: Superoxide dismutase
SS: Soluble carbohydrates
ST: Seed types
SVI-1: Seed vigor index-1
SVI-2: Seed vigor index-2
TPC: Total phenolic compounds
